# Development and Analysis of Graphene-Sheet-Based GaAs Schottky Solar Cell for Enriched Efficiency

**DOI:** 10.3390/mi14061226

**Published:** 2023-06-10

**Authors:** L. Kholee Phimu, Rudra Sankar Dhar, Khomdram Jolson Singh, Amit Banerjee

**Affiliations:** 1 Department of Electronics and Communication Engineering, National Institute of Technology Mizoram, Aizawl 796012, India; 2 Department of Electronics and Communication Engineering, Manipur Institute of Technology, Canchipur, Imphal 795003, India; 3 Microsystem Design-Integration Lab, Physics Department, Bidhan Chandra College, Asansol 713303, India

**Keywords:** external quantum efficiency, graphene, power conversion efficiency, Schottky barrier solar cell (SBSC), TCAD

## Abstract

Comparative studies of the 2D numerical modelling and simulation of graphene-based gallium arsenide and silicon Schottky junction solar cell are studied using TCAD tools. The performance of photovoltaic cells was examined while taking parameters, such as substrate thickness, relationship between transmittance and work function of graphene, and n-type doing concentration of substrate semiconduction. The area with the highest efficiency for photogenerated carriers was found to be located near the interface region under light illumination. The significant enhancement of power conversion efficiency was shown in the cell with a thicker carrier absorption Si substrate layer, larger graphene work function, and average doping in a silicon substrate. Thus, for improved cell structure, the maximum *J_SC_* = 4.7 mA/cm^2^, *V_OC_* = 0.19 V, and fill factor = 59.73% are found under AM1.5G, exhibiting maximum efficiency of 6.5% (1 sun). The EQE of the cell is well above 60%. This work reports the influence of different substrate thickness, work function, and N-type doping on the efficiency and characteristics of graphene-based Schottky solar cells.

## 1. Introduction

Due to graphene’s unique structure and characteristics, a single atomic layer has attracted significant attention, such as high mobility, low resistivity, and band gap [1]. Graphene has been created as ultrathin sheets made of a few atomic layers through mechanical exfoliation or CVD (chemical vapour deposition) and can be shifted to many substrates; thus, it will open up a large range of potential applications, including smart composites, photo sensors, and high-performance electronic devices [2]. Specifically, the graphene layer is a major material for use in the production of effective solar cells due to its exceptional combination of optical transparency and high electrical conductivity in the visible and near-infrared spectrum [3,4]. On various substrates, such as Si [5], CdS [6], CdSe [7], and GaAs [8], graphene-based Schottky junction solar cells have been produced in recent years. A Schottky junction was successfully formed on n-type GaAs by Wenjing et al., producing a power conversion efficiency of 1.95% [9]. GaAs has more radiation resistance [10] than the Si substrate, which is most frequently used, and has a direct band gap [11], which makes it suitable for highly efficient solar cells for both terrestrial and space applications. However, in order to increase solar cell efficiency, the band parameter must be studied, and various thicknesses of the structure must be optimised. We, therefore, optimised the thickness of the GaAs substrate with a graphene layer in SILVACO TCAD in this paper, and the results were confirmed using published experimental data.

**Figure 1:**
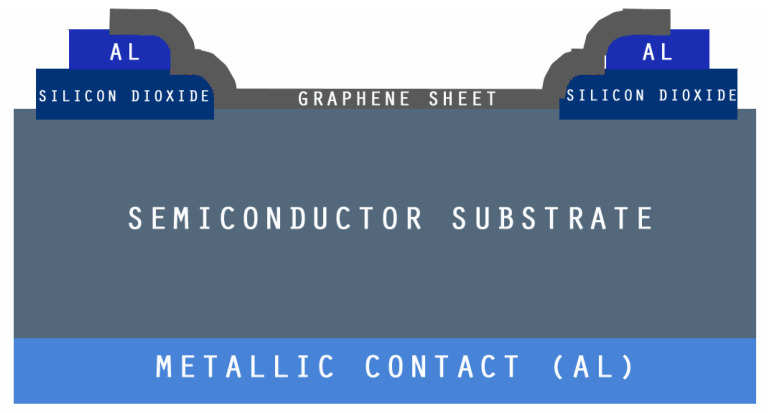
Proposed structure of Schottky graphene solar cell.

**Figure 2:**
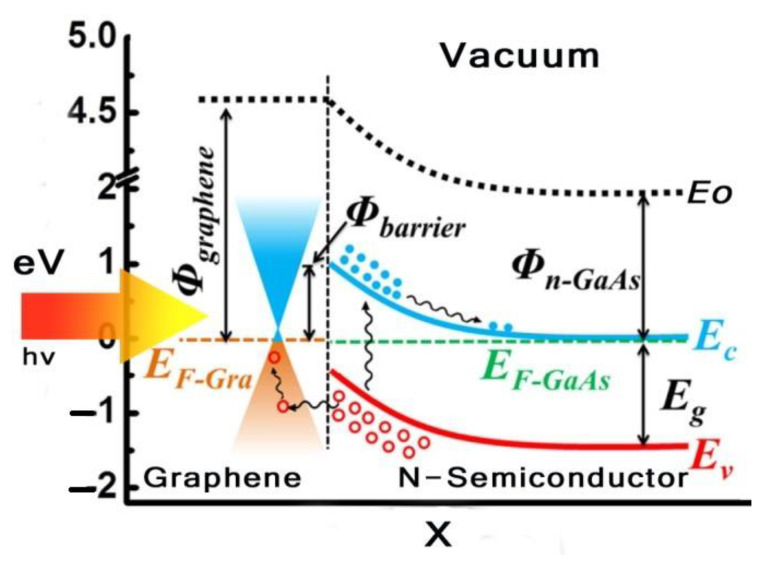
Energy band diagram of graphene solar cell.

The proposed graphene structure is shown in Figure 1. It consists of three regions. The ability to create graphene-on-silicon Schottky solar cells at room temperature opens up a wide range of applications for light gathering and conversion with the benefits of environmental friendliness and lower cost [12]. In this design, the graphene sheet performs dual roles for separation of holes/electrons as an active layer and as a carrier medium for transportation, in addition to acting as a transparent electrode for the transmission of light [13]. Despite the fact that the initial energy conversion efficiency is only 1.65% [14], the performance of graphene silicon Schottky cells was improved using silicon nano-array substrate adoption [15], chemical doping [5], and a graphene/P3HT/silicon configuration [16], ranging from 1.96% up to 10.3%. Some of the critical parameters, such as surface charge recombination, work function, and graphene conductivity, played a key role in establishing the performance of the device. Still, there has not been much research conducted on Schottky barriers. Thus, considerably more consideration and study are needed for the use of graphene in Schottky solar cells. Figure 2 shows an energy band diagram of graphene solar cells.

The graphene GaAs and silicon junction solar cell are numerically simulated in this work using standard TCAD tools. It uses a typical method for materials such as amorphous, including state model of continuous density, auger recombination mechanisms, and Schottky, to solve continuity, Poisson, and current density equations. The dependence of these optical parameters with the photon energy has been included, taking into account the thickness of the substrate, work function of graphene, doping level, and their impact on cell efficiency.

## 2. Modelling Semi-Transparent Top-Layer Graphene in Atlas

Due to the fact that graphene is a novel material, it is not yet included in the SILVACO Atlas material library. As a result, in order to develop a reliable and exact model of the graphene film, the initial definition of the layer used 4H-SiC as the base material, which was then modified to give the metallic material properties and match experimental sheet resistance values [17]. First, the material 4H-SiC model was made entirely transparent without changing its optical characteristics. Next, the graphene transmittance was entered by creating an .nk file for graphene from the obtained value [18]. With 15,000 cm^2^/Vs of carrier mobility [19], graphene is described as the Fermi distribution, and band gap, effective masses, and thickness values of 10 nm were changed to make sure they matched the experimental findings. Table 1 is a list of the simulation parameters that the Atlas tool utilized for this cell [20].

## 3. Modelling Graphene-Based Solar Cells

**Figure 3:**
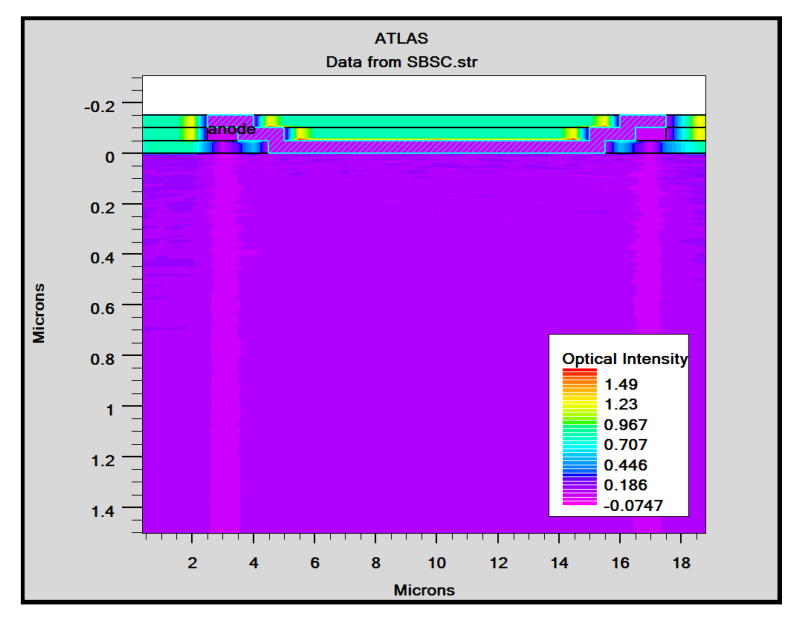
Optimized optical intensity in Si solar cell.

**Figure 4:**
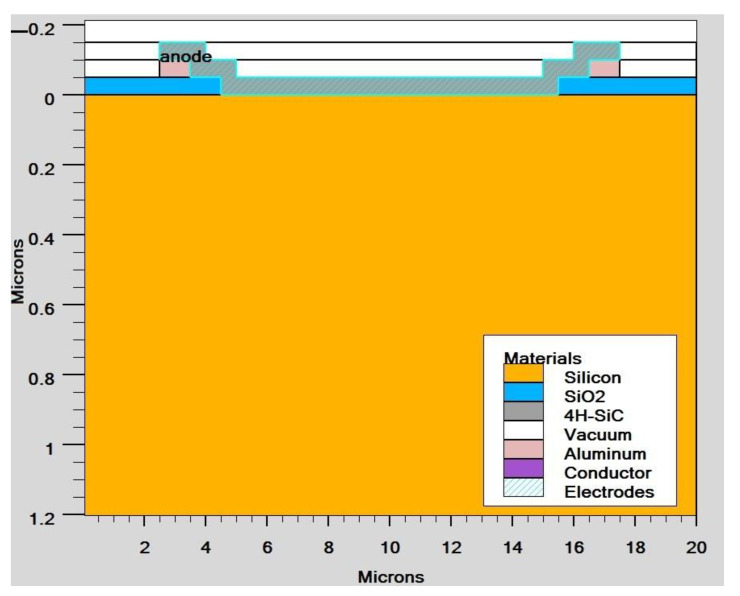
Solar cell cross-section view of graphene Si.

**Figure 5:**
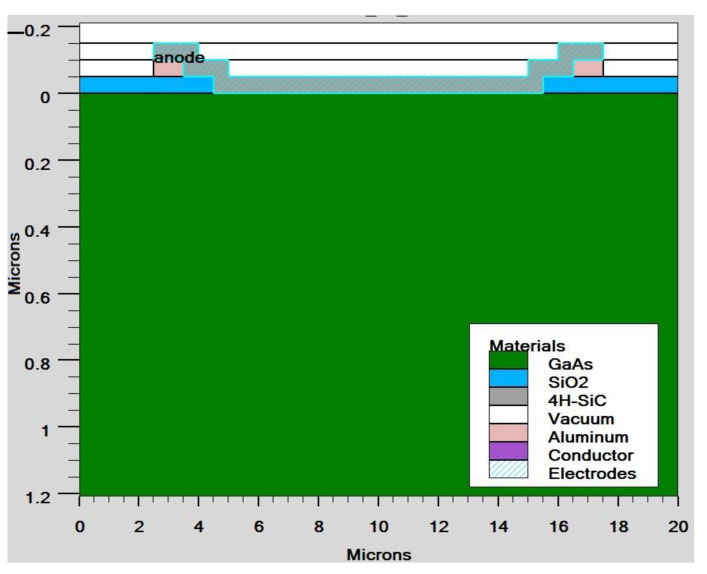
Solar cell cross-section view of graphene GaAs.

With the use of TCAD, including the optical intensity, as shown in Figure 3, and using parameters from Table 1, the user of ATLAS can choose from a number of physics models to compute recombination and carrier mobility. We utilized the following models in our design for our analysis. The doping-dependent low-field mobilities of holes/electrons in the cell at 300 K were modelled. The recombination models utilized were the Optical Recombination (OPTR) and the Schottky–Read–Hall (SRH) recombination models. As already stated, Figure 4 shows the cross section of a graphene Si solar cell that was modelled in TCAD software. The device is made up of three areas, from bottom to top, namely the silicon substrate, the SiO_2_ window, and the graphene layer. Here, an oxide window was used to coat the silicon substrate with a 10 nm thick layer of graphene. Figure 5 shows the detailed top layer of the cell.

Most studies show that the heterojunction device for this structure is fabricated from a transferred chemical vapour deposition graphene layer on silicon in order to avoid expensive deposition methods and complicated processing [21,22]. In essence, the Schottky junction can be created using any semiconductor with a specific metal, provided the work function differences are sufficiently large and the carrier densities are moderate. The calculations show that graphene sheets and silicon create a Schottky junction, which is advantageous for creating a sizable built-in field [14]. The photoexcited holes and electrons are produced in the silicon substrate when it is illuminated, and they are subsequently separated at the Schottky junction through the built-in electric field. The bottom electrodes gather electrons/holes, leading to a photovoltaic reaction. The thermionic emission model can be used to describe the Schottky junction’s non-linear I-V property [23]:

(1) $$I_{o}=\alpha B^{*}\;T^{2}\exp(-e\varphi_{Bn}/kT)$$

where α is area cell, B* is effective Richardson constant, $\varphi_{Bn}$ is metal–semiconductor (n-type), *k* is Boltzmann’s constant, and *T* absolute temperature.

(2) $$\varphi_{B}=\varphi_{G}-\chi ,\;\mathrm{for}\;\mathrm{n-type}\;\mathrm{semiconductor}$$

Additionally,

(3) $$\varphi_{B}=E_{G}-\varphi_{G}+\chi ,\;\mathrm{for}\;\mathrm{p-type}\;\mathrm{semiconductor}$$

where $\varphi_{G}$ = graphene work function, χ = electron affinity, *E_G_* = energy gap semiconductor.

## 4. Results and Discussion

**Figure 6:**
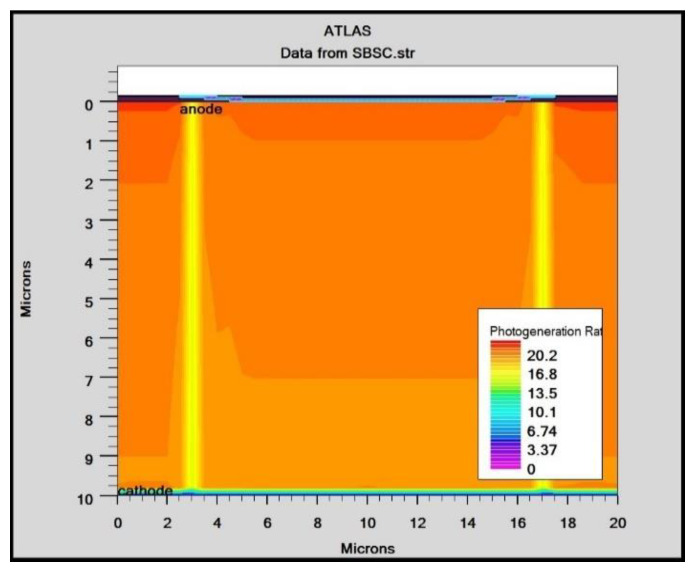
Photogeneration rate in Si solar cell.

**Figure 7:**
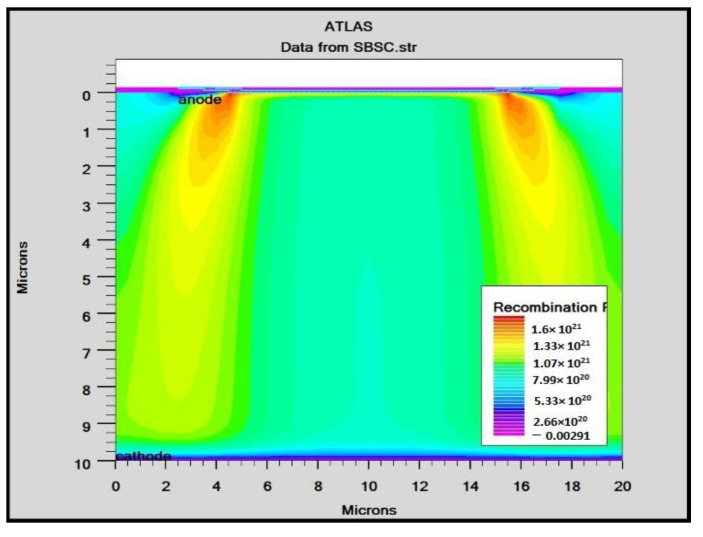
Recombination rate in Si Solar cell.

**Figure 8:**
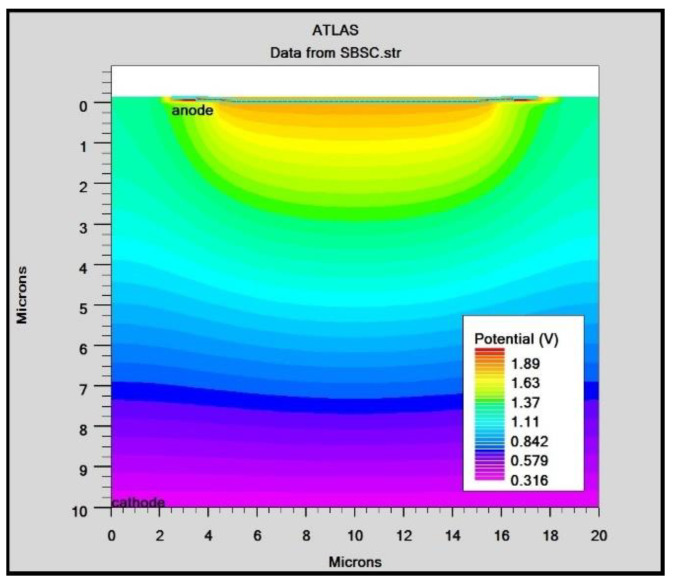
Potential of Si solar cell.

**Figure 9:**
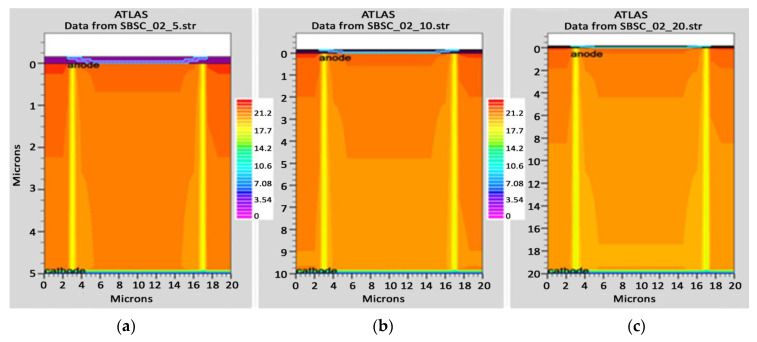
Photogeneration rate of Si cell: (**a**) 5 µm, (**b**) 10 µm, (**c**) 20 µm.

**Figure 10:**
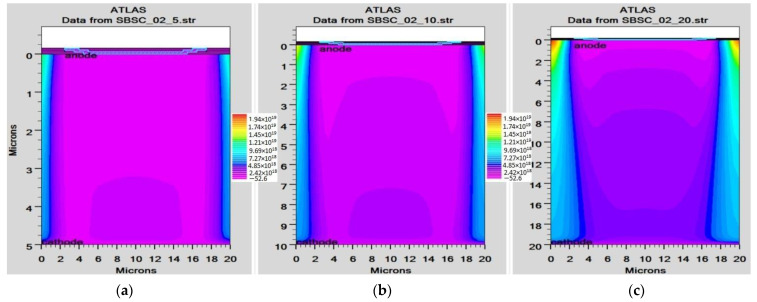
Electric field of Si cell: (**a**) 5 µm, (**b**) 10 µm, (**c**) 20 µm.

**Figure 11:**
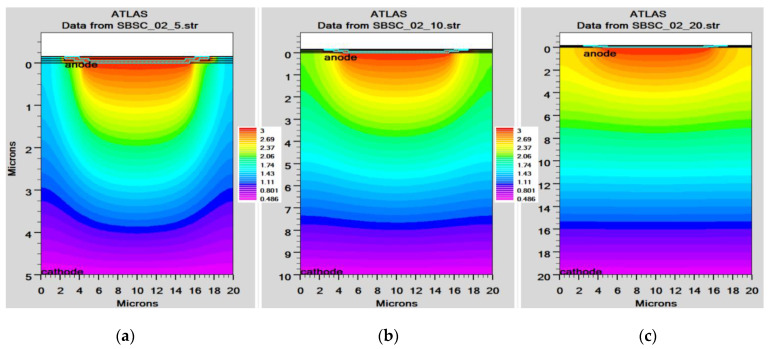
Potential of Si cell: (**a**) 5 µm, (**b**) 10 µm, (**c**) 20 µm.

In order to mimic the global terrestrial sunshine, the modelled device was illuminated with AM1.5G solar spectrum, which is taken into account by LUMINIOUS 3D for modelling sunlight in SILVACO Atlas [24]. The light could be absorbed in the barrier layer and inside the semiconductor. The photogeneration rate is provided by $G=\eta_{0}\frac{P\lambda }{hc}\alpha e^{-\alpha y}$, where P represents the total effect of the ray path’s absorption, reflection, and transmission losses, *y* is the ray for the provided relative distance, α is the computed absorption coefficient for every combination value of (*n*, *k*), *λ* is wavelength, *h* is Planck’s constant, c is the speed of light, and *η*_0_ is the internal quantum efficiency, denoting the carrier number produced per photon per pair. It can be seen from Figure 6 that the highest efficient absorption area is around 0.1 µm from the surface connection and it not only increases the light absorption length but is also concentrated to the light field, which results in enhanced excitation of photon-induced carriers. The recombination rate is also higher at the interface region, as indicated by the result shown in Figure 7. Figure 8 shows the potential developed inside the cell, indicating that maximum potential is developed in the anode vicinity and that there is a greater collection of charge. Figure 9, Figure 10 and Figure 11 show a comparison of different thicknesses for the photogeneration rate, electric filed, and potential, respectively. Figure 9a–c show the photogeneration rate of Si cells under AM1.5 (sun) with depths of absorption, which were examined at 5 µm, 10 µm, and 20 µm. The semiconductor and barrier could both absorb the light. Figure 9c shows that 20 µm thickness is more than sufficient for full-spectrum absorption because the intensity of the photogenerated carrier suddenly reduces in the deep area of the Si substrate. In Figure 10, as the number of solar cells rises, the electrical field also rises. In fact, an increase in the number of solar cells leads to an increase in the open-circuit photovoltage. The main aim of this paper is to enhance the conversion efficiency through a reduction in silicon matter. As a result, fewer solar cells must be utilised to generate the electrical field, which lowers the amount of semiconductor materials needed to make solar cells. Figure 10 gives the evolution of the electric field as a function of the solar cells. Figure 11 shows the potential developed inside the cell, showing that maximum potential is developed in the anode vicinity, indicating a greater collection of charge.

It is crucial to utilize *I_SC_* and *V_OC_* to establish how well a solar cell works because they affect how much power it can produce.

(4) $$V_{OC}=\frac{nKT}{q}ln\left(\frac{I_{L}}{I_{0}}+1\right)$$

(5) $$FF=\frac{V_{OC}-Ln\left(V_{OC}+0.72\right)}{V_{OC}+1}=\frac{I_{m}V_{m}}{I_{SC}V_{OC}}$$

*I_SC_* and *V_OC_* utilisation at maximum power is measured via the fill factor. The efficiency can be expressed using *FF* as:

(6) $$\eta =\frac{V_{OC}I_{SC}FF}{P_{in}}=\frac{P_{max}}{1000\;\left[{\mathrm{Wm}}^{-2}]\times CellArea\;[m^{2}\right]}$$

These are the performance metrics that we employed in our research for evaluating solar cells.

### 4.1. GaAs and Si Thickness Effect

**Figure 12:**
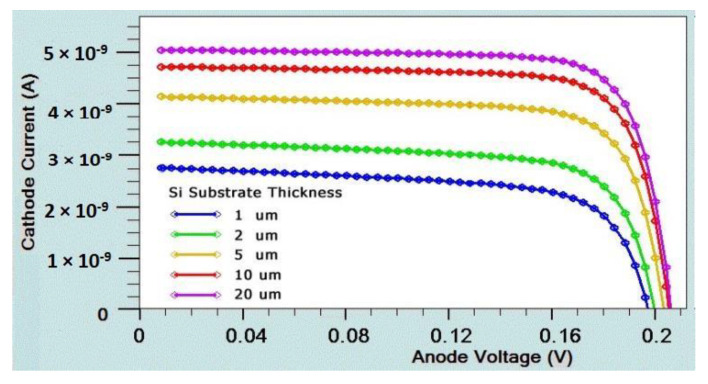
I-V curve of Si substrate with different thicknesses.

**Figure 13:**
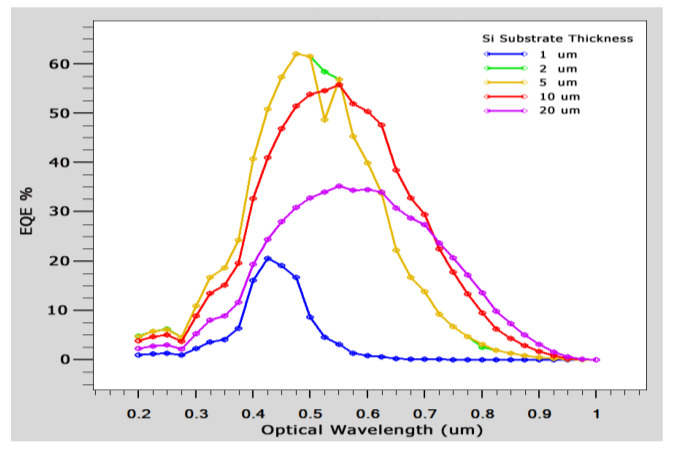
EQE of solar cell vs. different Si thicknesses.

The Si crystal substrate is illuminated with an orientation <100>. For photogenerated carrier intensity, graphene functions as a transparent electrode. The difference between the work functions was built using a Schottky junction. We computed the I-V curves and external quantum efficiency (EQE), as illustrated in Figure 12 and Figure 13, respectively, to examine the impact of silicon thickness on solar cell performance. Since silicon has an indirect band gap, it works with longer wavelengths and results in higher quantum efficiency. When silicon crystal thickness decreases, long-wavelength light passes through the device and lowers the IQE. On the other hand, the enhanced recombination carrier rate on the back electrode causes a significant increase in the dark current. Because the silicon substrate is thicker, it is discovered that the efficiency of the device ranges from 2.37% to 5.99% (given in Table 2).

### 4.2. Graphene Work Function Effect

The difference between silicon *χ* and graphene *ф_G_* is related to the barrier height *ф_B_*, as discussed in Section 3. Therefore, a higher work function will enhance the *ф_B_*, which will make the built-in potential *V_bi_* increase according to the equation, *V_bi_* = *ф_B_* − *V_n_*, where *V_n_* is the distance between *Ec* and *Ef* in silicon. Consequently, an enhanced graphene work function results in an increase in *V_bi_* that corresponds to the upper limit of *V_oc_*. As shown in Table 3, the built-in potential *V_bi_* increases monotonically along with the *FF* and power conversion efficiency. This result is inconsistent with Y.F. Li’s group’s experimental findings [25].

### 4.3. GaAs and Silicon n-Type Doping Effect

The ability of silicon’s N-type doping to raise the photogeneration carrier intensity and energy level of Fermi is well recognised. Due to the larger barrier height compared to a substrate made of pure silicon, both *V_OC_* and *I_SC_* increase for doping concentrations below 3 × 10^15^ cm^−3^. When the doping concentration exceeds 3 × 10^15^ cm^−3^, the cell’s performance is radically different. The mechanism for electron emission could be the origin of this phenomenon. The emissions of a carrier from silicon to graphene are dominated by the tunnelling emission process rather than the thermionic emission mechanism. As a result, there is a slight decrease in the open-circuit voltage. Second, a high doping level reduces the photogeneration carriers’ lifetime, particularly for light that has a long wavelength. These losses in the carrier collection result in a reduction in *I_SC_*. Thirdly, as indicated in Table 4, the 3 × 10^16^ cm^−3^ doping level has the largest fill factor, despite having a lower power conversion efficiency than the other two doping concentrations. Therefore, the optimal efficiency is found at a moderate doping level of 3 × 10^15^ cm^−3^.

**Figure 14:**
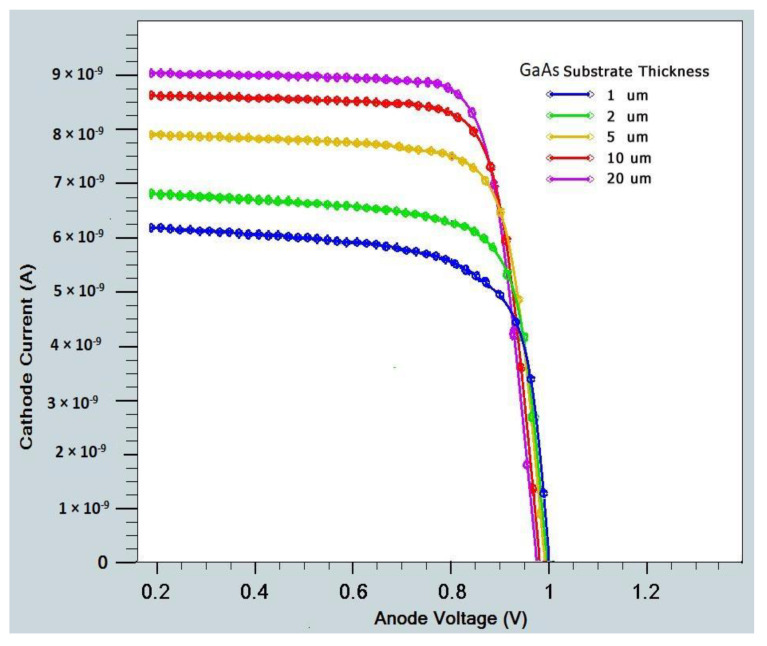
I-V curve of GaAs substrate with different thicknesses.

**Figure 15:**
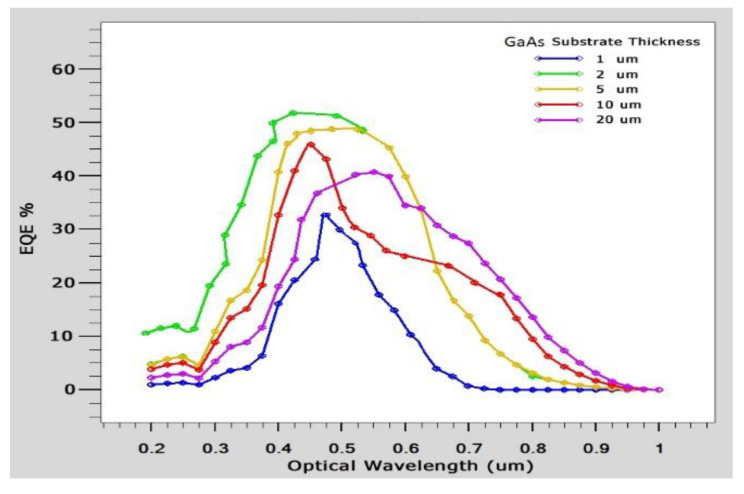
EQE of solar cell vs. different GaAs thicknesses.

In order to improve the design, it is also necessary to identify different performance parameters. A solar cell I-V curve with varying substrate thicknesses is depicted in Figure 12. We determine the current–voltage curves and external quantum efficiency (EQE) for GaAs, as shown in Figure 14 and Figure 15, respectively. It is found that Si EQE has well above 60% and GaAs EQE has 50%, which is optimized at the (2–5) µm substrate thickness due to the high photogeneration rate found at this substrate thickness.

**Figure 16:**
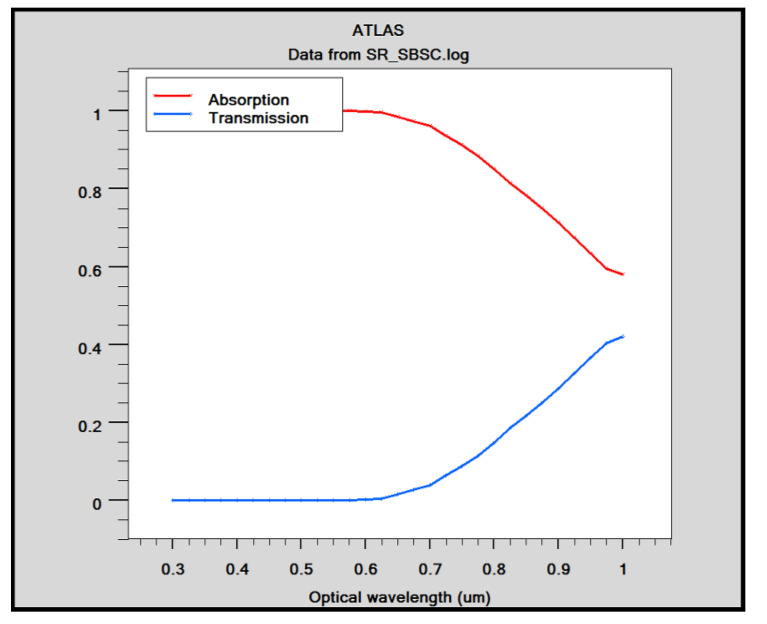
Absorption and transmission co-efficient of solar cells.

The absorption and transmission co-efficient of the cell is depicted in Figure 16. The proposed cell works effectively in a wavelength range of 300 to 700 nm. After 700 nm, the efficiency starts decreasing due to an increase in transmittance and less absorptivity of photons, as shown in Figure 16. Most of the spectrum is found to be utilized in the generation of the carrier in the optimal 10 µm thickness Silicon substrate. Equation (7) can be used to determine *J_SC_* using the measured *EQE*.

(7) $$J_{SC}=q\int^{\text{​}}F\left(\lambda \right)EQE\left(\lambda \right)d\lambda$$

where *F*(*λ*) = intensity spectrum of AM1.5G sunlight, and *q* is electron charge. It is found that *EQE* is well above 60%, which is an indication of an effective cell structure. Table 5 provides a tabular comparison that demonstrates that the Si substrate is significant when compared with existing results. Through comparison with the existing results, it is found that graphene-based Schottky solar cells with Si and GaAs as substrates have increased efficiency of GaAs (4.74%) and Si (5.99%) over conventional Schottky junction solar cells. 

## 5. Conclusions

Silvaco ATLAS software was used to model graphene-based GaAs solar cells and graphene-on-Si Schottky junction solar cells in a unique way for photovoltaic applications. Numerical simulation in two dimensions was used to analyze the output performance. Additionally, we thoroughly examined the performance vs. various graphene work functions, substrate thicknesses, and N-type doping concentrations. The findings demonstrate that greater graphene work function, adequate absorption thickness, and mild silicon doping are superior for increasing the power conversion efficiency. Further analysis revealed that the anode’s vicinity experienced the development of the highest potential, which leads to improved charge collection and an improvement in the solar cell’s overall performance. It is possible to research, design, and construct various structures of graphene-based solar cells using this remarkable optimization technique and data. The conversion of the emission electron mechanism from the thermionic mechanism to the tunnelling emission mechanism is due to the implementation of high-level doping, resulting in maximum *FF* but lower efficiency. For GaAs, under low illumination, the majority of the carriers created in the region reside relatively near to the interface. When compared to graphene’s lower transmittance and greater work function regarding cell performance, the work function is effective for increasing the cell performance. GaAs with moderate n-type doping has a significant increase in power conversion efficiency; however, this form of device is significantly more sensitive to open-circuit voltage than short-circuit current. Therefore, Si has maximum conversion efficiency in terms of thicker absorption, larger graphene work function, and average doping in semiconductor substrates. As a result, it can be concluded from this work that graphene can function as a semi-transparent charge collector electrode and Schottky junction with a thermionic emission phenomenon for improved efficiency in SBSC solar cells.

## Figures and Tables

**Table 1:** Material utilized in this numerical simulation.

Essential Layer Properties	ATLAS Identifier	Material Layer		
		Graphene	Substrate	
		4H-SiC	GaAs	Silicon
Band gap layer E_g_ (eV)	E_G_	0.0	1.42	1.08
Relative permittivity, ε_r_ (F cm^−1^)	Permittivity	25	13.1	13.5
Electron affinity X_e_ (eV)	Affinity	5.8	4.07	4.17
Mobility, µ_p_ (cm^2^/V s)	MUP	15,000	400	500
Mobility, µ_n_ (cm^2^/V s)	MUN	15,000	8000	1000
Effective density of states Nc (cm^−3^)	NC300	3 × 10^17^	4 × 10^17^	2.8 × 10^19^
Effective density of states Nv (cm^−3^)	NV300	3 × 10^17^	7 × 10^18^	1.0 × 10^19^

**Table 2:** Performance of cell with substrate thickness (under n doping 3 × 10^16^ cm^−3^ and work function 4.8).

Thickness (µm)	*J_SC_* (mA/cm^2^)		*V_OC_* (V)		*FF* (%)		Efficiency (%)	
	GaAs	Si	GaAs	Si	GaAs	Si	GaAs	Si
1	6.10	2.76	1.00	0.29	51	28.69	3.29	2.37
2	6.80	3.26	0.97	0.29	51	31.88	5.03	3.11
5	7.90	4.14	0.94	0.18	49	58.08	4.74	4.51
10	8.40	4.72	0.93	0.19	45	60.21	4.58	5.47
20	9.00	5.05	0.91	0.19	45	60.95	4.58	5.99

**Table 3:** Performance of cell with graphene work function (under optimal 10 µm si-thickness).

Graphene Work Function	*J_SC_* (mA/cm^2^)		*V_OC_* (V)		*FF* (%)		Efficiency (%)	
	GaAs	Si	GaAs	Si	GaAs	Si	GaAs	Si
4.4	6.10	4.72	1.00	0.18	51	50.34	3.29	4.9
4.6	6.10	4.72	0.97	0.16	51	48.21	5.03	5.3
4.8	6.10	4.72	0.94	0.19	49	60.21	4.74	5.4

**Table 4:** Performance of cell with doping concentrations (under optimal 10 µm si- thickness).

Doping of n-Type Effect (/cm^−3^)	*J_SC_* (mA/cm^2^)		*V_OC_* (V)		*FF* (%)		Efficiency (%)	
	GaAs	Si	GaAs	Si	GaAs	Si	GaAs	Si
3 × 10^14^	6.10	4.7	1.38	0.20	68	57.04	9.54	5.65
3 × 10^15^	6.10	4.7	1.37	0.19	66	59.73	9.52	6.50
3 × 10^16^	6.10	4.3	1.36	0.18	67	60.21	9.50	5.47

**Table 5:** Comparison of various factors, such as substrate thickness, work function, and doping concentration, with existing device.

	**GaAs Thickness (µm)**	**Work Function (ev)**	**N-Type Doping (/cm^3^)**	***J_SC_* (mA/cm^2^)**	***V_OC_* (V)**	***FF* (%)**	**Efficiency (%)**
GaAs junction solar cell [26]	5	4.8	1 × 10^14^	7.966	0.301	49	1.518
Our proposed work	5	4.8	3 × 10^16^	6.10	0.94	49	4.74
	**Si Thickness (µm)**	**Work Function (ev)**	**N-Type Doping (/cm^3^)**	***J_SC_* (mA/cm^2^)**	***V_OC_* (V)**	***FF* (%)**	**Efficiency (%)**
Si junction solar cell [27]	20	4..8	1 × 10^17^	5.72	0.158	58	0.874
Our proposed work	20	4..8	3 × 10^16^	5.05	0.19	60.95	5.99

## Data Availability

Not applicable.
